# An Unusual Pulmonary Aspergillus Infection: Endobronchial Aspergilloma

**DOI:** 10.7759/cureus.33587

**Published:** 2023-01-10

**Authors:** Ashok Kuwal, Rishabh Kochar, Rishabh Agarwal

**Affiliations:** 1 Pulmonary Medicine, Dr. Sampurnanand Medical College and Associated Group of Hospitals, Jodhpur, IND; 2 Pulmonary Medicine, All India Institute of Medical Sciences (AIIMS), Jodhpur, IND; 3 Pulmonary Medicine, Pacific Institute of Medical Sciences, Udaipur, IND

**Keywords:** haemoptysis, endobronchial aspregilloma, endobronchial mass, pulmonary aspergillosis, aspergillus

## Abstract

Endobronchial aspergilloma (EBA) is an extremely rare presentation of pulmonary involvement of Aspergillus. It is a noninvasive form of *pulmonary aspergillosis* where the fungus overgrows and colonizes the bronchial lumen. The patient may present with chronic cough, dyspnea, hemoptysis, or wheezing. The diagnosis is usually incidental when bronchoscopy is performed to evaluate the cause of hemoptysis or radiological abnormalities. Here, we report a case of a middle-aged female who presented with hemoptysis and right middle lobe collapse and was subsequently diagnosed to have EBA on bronchoscopy with endobronchial biopsy. Although EBA is rare, it should be considered as a differential in the evaluation of endobronchial mass lesions.

## Introduction

Aspergillus spp. is a ubiquitous fungus responsible for a broad spectrum of lung diseases, depending on the patient's immune status and underlying lung disease. Pulmonary involvement of aspergillosis can be in the form of pulmonary aspergilloma, allergic bronchopulmonary aspergillosis (ABPA), chronic necrotizing pulmonary aspergillosis, or invasive pulmonary aspergillosis [1].

In patients with preexisting lung disease, Aspergillus can colonize a preexisting cavity to form an aspergilloma (fungus ball). ABPA is a type of hypersensitivity reaction to the presence of Aspergillus antigens and is seen predominantly in patients with underlying asthma. While invasive pulmonary aspergillosis is a severe and fatal disease primarily observed in severely immunocompromised patients, chronic necrotizing pulmonary aspergillosis is characterized by a local hyphal invasion into the lung tissue in patients with chronic lung disease and underlying diabetes mellitus, corticosteroid therapy, or malnutrition.

An endobronchial aspergilloma (EBA) is an unusual presentation of pulmonary aspergillosis; it is characterized by the growth of the Aspergillus species into the bronchial lumen [2,3]. Here, we present a case of a female presenting with streaky hemoptysis and radiological abnormality consistent with endobronchial growth. Bronchoscopy with endobronchial biopsy led to the diagnosis of EBA.

## Case presentation

**Figure 1:**
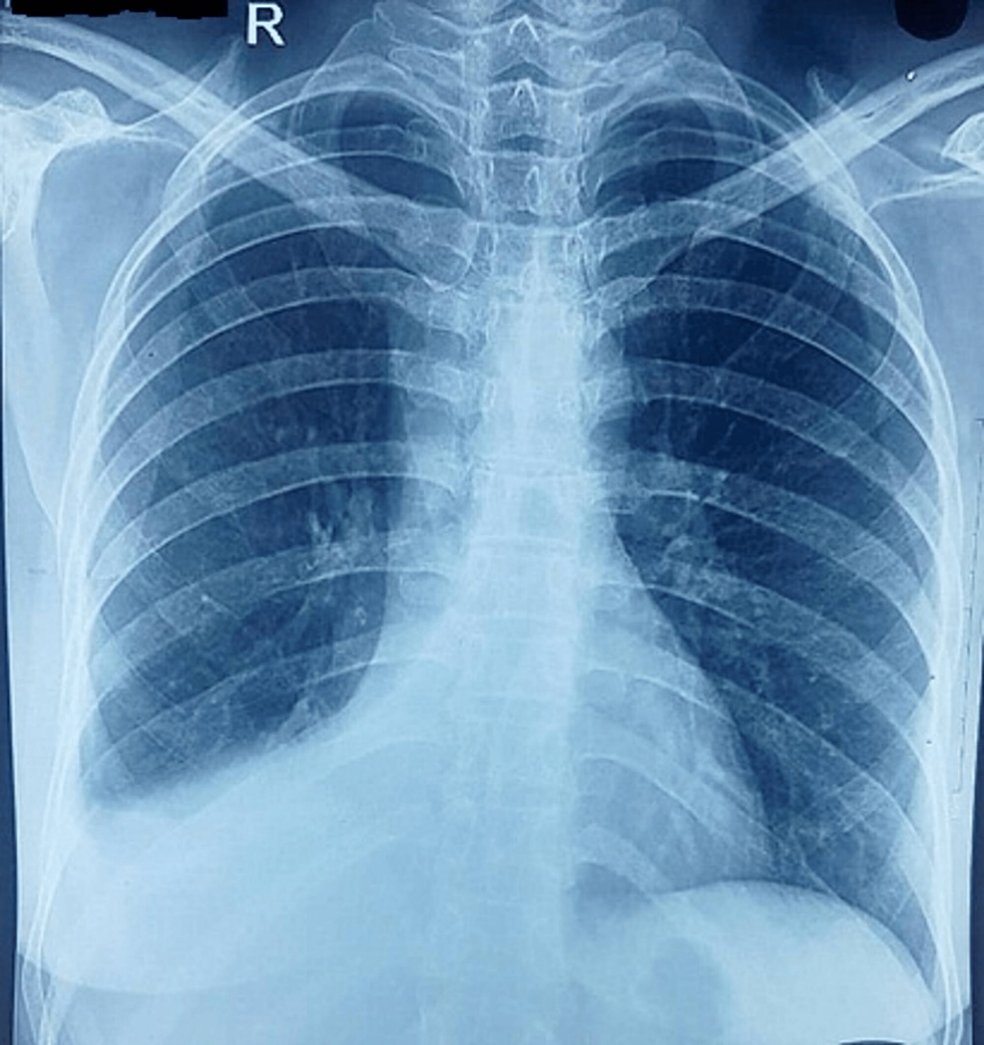
Chest radiograph showing elevated right hemidiaphragm and silhouetting of the right heart border - suggestive of the right middle and lower lobe collapse.

**Figure 2:**
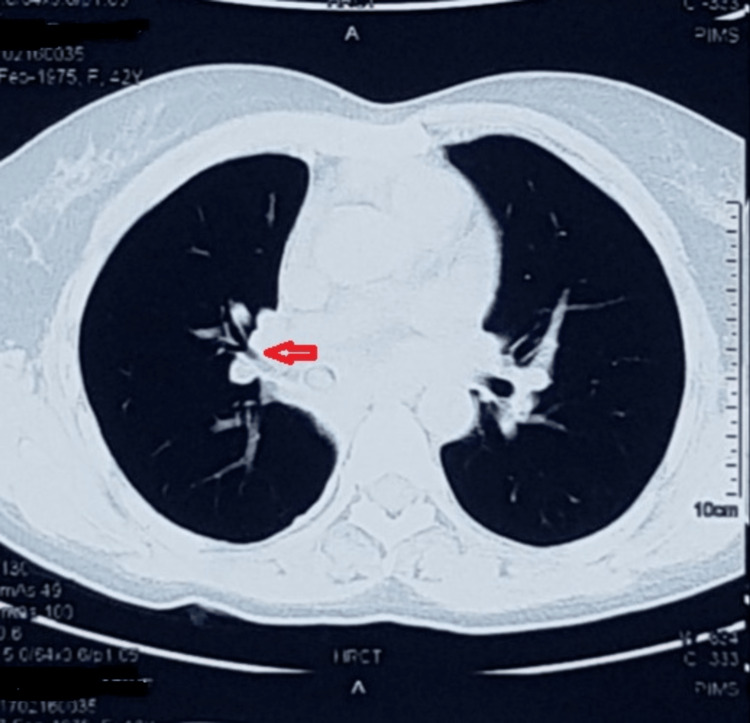
HRCT of the chest showing an endoluminal mass in the right bronchus intermedius. HRCT, high-resolution computed tomography

A 42-year-old female presented with complaints of cough with blood in her sputum for two days, with a history of back pain, generalized weakness, and chest pain for one year. Based on her history and radiology, the patient was empirically treated with antitubercular therapy for 11 months at the primary care center. The patient presented to our center because her symptoms had not resolved on antitubercular therapy, and she had new onset hemoptysis. The patient was a nonsmoker and nonalcoholic and had no history of hypertension, diabetes mellitus, asthma, or other chronic illness. General physical examination was unremarkable. On auscultation, the intensity of breath sounds was reduced over the right anterior chest, axilla, and infra-scapular areas. Chest radiograph showed an elevated right hemidiaphragm with silhouetting of the right heart border, which was suggestive of right middle and lower lobe collapse (Figure 1). High-resolution computed tomography (HRCT) of the chest revealed an abrupt cutoff of the right bronchus intermedius (Figure 2) with a collapse of the right middle and lower lobes.

**Figure 3:**
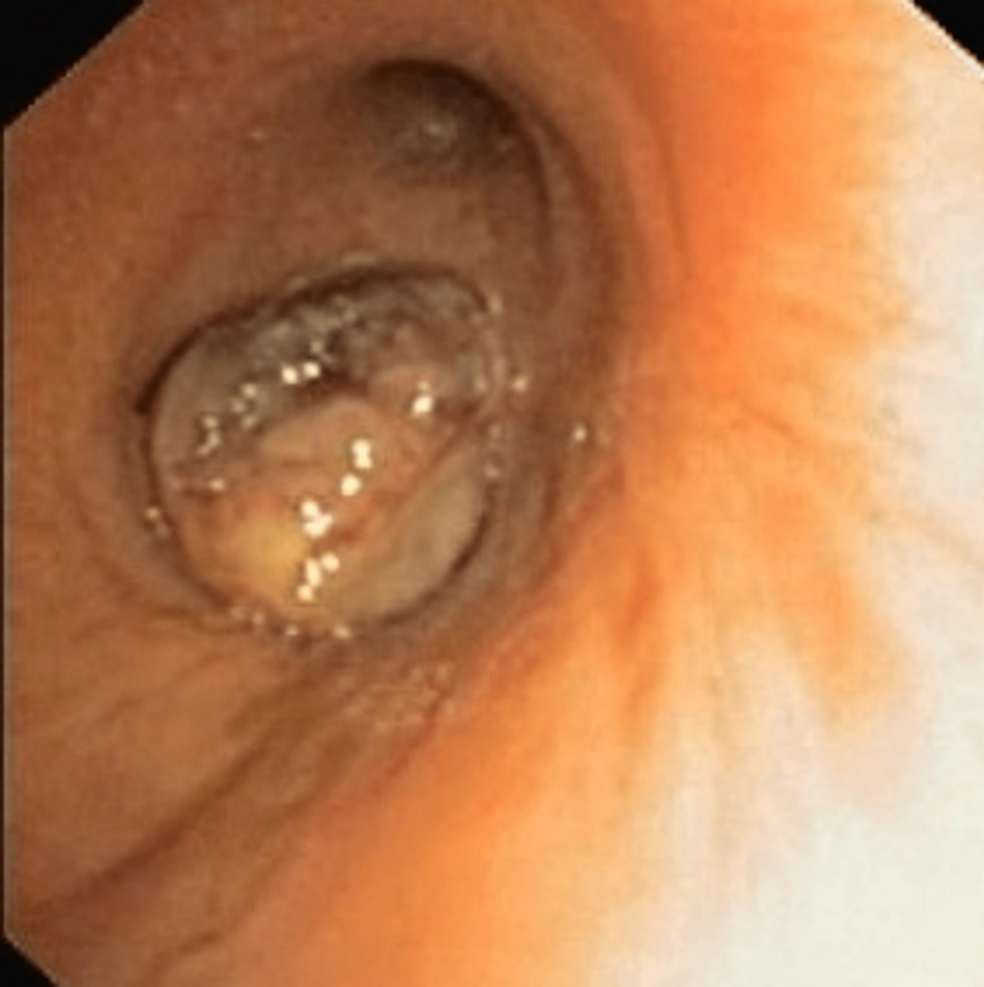
Bronchoscopic image showing fleshy, lobulated endobronchial mass completely occluding the right bronchus intermedius.

**Figure 4:**
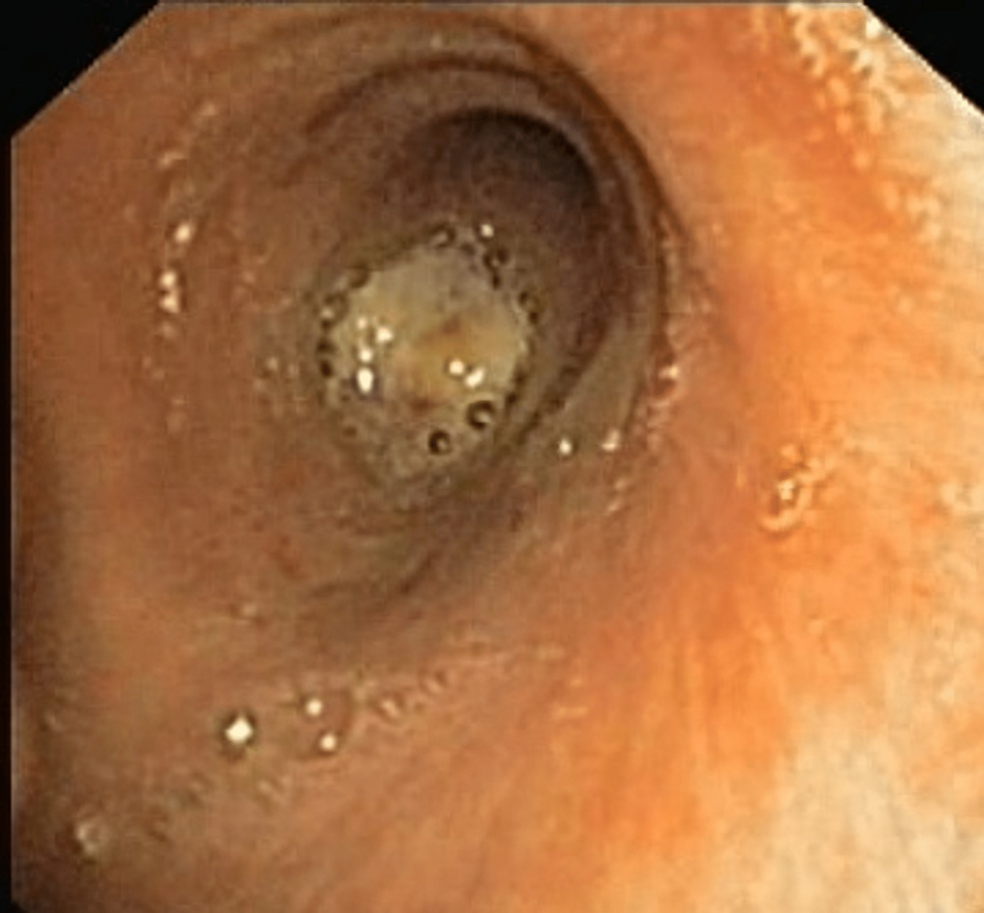
Follow-up bronchoscopy after six weeks of voriconazole showing a reduction in the size of the endobronchial mass.

Diagnostic bronchoscopy showed a large lobulated mass at the opening of the right bronchus intermedius near total occlusion of the lumen (Figure 3). Endobronchial biopsy revealed massive areas of necrosis, abundant inflammatory cells comprising predominantly neutrophils, and a large number of septate fungal hyphae. The possibility of an underlying malignancy was still not ruled out, so the bronchoscopy was repeated, and more endobronchial biopsies were taken from the deeper aspect of the lesion. However, histopathological examination revealed the same findings as previous, with no evidence of malignancy, and the culture of the biopsy specimen yielded colonies of Aspergillus fumigatus. A diagnosis of EBA was made, and voriconazole was initiated. The patient improved clinically, and a repeat bronchoscopy after six weeks revealed a considerable decrease in the size of the endobronchial lesion (Figure 4).

## Discussion

EBA is a rare clinical entity caused by the endoluminal growth of Aspergillus in patients with or without preexisting lung disease. The pulmonary manifestations of Aspergillus were initially described by Hinson et al. [1]. They classified them as saprophytic, allergic, and septicemic based on the patient’s immune status [1]. EBA has been extensively neither described in the literature nor classified as per the current classification of Aspergillus infections involving the lungs and the airways. Aspergilloma is classically a space-occupying lesion with overgrowth of fungus, necrotic tissue, and mucus within the nidus of a preformed cavity or previously damaged areas of the lung from past disease or procedure - usually tuberculosis, postsurgery stump, or in patients with underlying malignancy. It is the most common form of lung involvement due to Aspergillus. Aspergilloma usually grows without invading the host tissue and does not cause local tissue erosion [4].

Although intrapulmonary aspergilloma is a common and frequently reported entity, intrabronchial aspergilloma is relatively rare, with very few case reports. Not much is known about the pathophysiology and management of EBA. The most common presenting symptoms of EBA include cough, hemoptysis, chest pain, and dyspnea, with hemoptysis as the most common symptom [3]. Most of the cases of EBA were reported in patients with a history of structural airway disease or an immunocompromised state. Indeed, the first reported EBA was seen in three AIDS patients and was termed obstructing bronchial aspergilloma [5]. In a case series by Ma et al., all 10 patients with EBA had a predisposing risk factor - seven patients had pulmonary tuberculosis, two patients had lung cancer, and one had a suspected foreign body in the bronchus [3]. On the contrary, our patient had no underlying lung disease or an immunocompromised state. This makes the present case even rare as the patient had no predisposing condition for nidus formation and fungal colonization. EBA is also associated with lung parenchymal involvement, as reported in two cases by Ma et al. [3], but our patient had no pulmonary involvement apart from the endobronchial mass.

The diagnosis of EBA is not straightforward on imaging. It can be frequently confused with other airway lesions such as foreign body, malignant growth, or as an incidental diagnosis while investigating a fungal ball in the lung [6]. The diagnosis of EBA can be made only after bronchoscopy and biopsy. CT of the chest may help characterize the mass lesion within the bronchus but does not lead to a definitive diagnosis. The lesions frequently masquerade as endobronchial tumors, and it is only after a biopsy that aspergillosis is diagnosed. As in this case, a biopsy from the deeper part of the lesion is required to rule out any underlying malignancy definitively [7]. The bronchoscopic appearance of EBA has been consistently reported as a round, whitish-to-yellow-colored mass, occasionally reddish brown, and found to be partially or completely obstructing the airway. Histopathological examination is necessary for a definitive diagnosis and to rule out any other associated endobronchial pathology before proceeding to treatment.

The optimal treatment strategy for EBA is still not known. Various treatment options like observation and partial or complete pneumonectomy have been suggested. Ma et al. favored a conservative approach, and most of their patients (8 out of 10) were not given any specific therapy as aspergilloma was presumed to be saprophytic colonization without invasion. They argued that systemic antifungals have poor penetration in the lung and that providing only symptomatic therapy was adequate [3]. Aggressive measures like systemic antifungals, bronchoscopy-guided resection, bronchoscopic drug instillation, inhaled antifungals, and partial or complete lung resection have been tried and were found to be effective in managing the disease [8]. Due to poor drug penetration, systemic antifungal therapy is not useful in cases with pulmonary aspergillomas [9]. Argento et al. treated two patients with airway obstruction due to aspergilloma with combination therapy of bronchoscopy-guided resection and systemic antifungals and reported good outcomes in both cases [10]. As our patient was without comorbidities and clinically stable, we opted for a conservative approach with antifungal with surgical intervention as a second option. Our patient showed a good response to systemic voriconazole, with symptomatic improvement within two weeks of initiating treatment and a reduction in the size of the endobronchial lesion on a six-week follow-up.

## Conclusions

EBA is a rare but possible differential in patients with endobronchial mass lesions. Clinical suspicion for EBA should be high if the patient with endobronchial growth is immunocompromised or has structural lung disease. The bronchoscopic appearance is noncharacteristic and frequently resembles an endobronchial carcinoid. As preexisting endobronchial masses may also be colonized with fungus, a definitive diagnosis is possible only after the biopsy from the deeper aspects of the lesion. Although no specific therapy is recommended, multiple modalities - watchful monitoring, medical therapy, bronchoscopic interventions, and surgery - have been tried with varying success rates. In clinically stable patients diagnosed with EBA, we suggest an initial trial of systemic antifungals before considering more invasive therapeutic options.
